# Effect of natural curcuminoids‐intercalated layered double hydroxide nanohybrid against *Staphylococcus aureus*,* Pseudomonas aeruginosa*, and *Enterococcus faecalis*: A bactericidal, antibiofilm, and mechanistic study

**DOI:** 10.1002/mbo3.723

**Published:** 2018-09-17

**Authors:** Buddhika Gayani, Ayomi Dilhari, Gayan Kanchana Wijesinghe, Sajeewani Kumarage, Gayani Abayaweera, Sameera R. Samarakoon, Inoka C. Perera, Nilwala Kottegoda, Manjula M. Weerasekera

**Affiliations:** ^1^ Department of Chemistry Faculty of Applied Sciences University of Sri Jayewardenepura Gangodawila, Nugegoda Sri Lanka; ^2^ Center for Advanced Materials Research Faculty of Applied Sciences University of Sri Jayewardenepura Gangodawila, Nugegoda Sri Lanka; ^3^ Department of Microbiology Faculty of Medical Sciences University of Sri Jayewardenepura Gangodawila, Nugegoda Sri Lanka; ^4^ Sri Lanka Institute of Nanotechnology, Nanoscience and Technology Park Pitipana, Homagama Sri Lanka; ^5^ Institute of Biochemistry Molecular Biology and Biotechnology University of Colombo Colombo Sri Lanka; ^6^ Department of Zoology and Environment Sciences Faculty of Science University of Colombo Colombo Sri Lanka

**Keywords:** antibiofilm, antimicrobial, curcuminoids, intercalated, layered double hydroxide

## Abstract

The study aimed to determine the antibacterial/antibiofilm effect and mechanism of interaction of curcuminoids‐intercalated Mg/Al layered double hydroxide (curcuminoids‐LDH) against three different bacteria. Antimicrobial effect of curcuminoids‐LDH nanohybrid was investigated against *P*. *aeruginosa*,* S*. *aureus*, and *E*. *faecalis* (for both standard strains and clinical isolates), using agar well diffusion method. Minimum inhibitory concentrations (MIC) of planktonic bacteria were determined using the broth microdilution method. MIC of biofilms (MBIC_50_) and killing time for 48 hr matured biofilms were determined by MTT (3‐(4,5‐Dimethylthiazol‐2‐yl)‐2,5‐diphenyltetrazolium bromide) assay. Scanning electron microscopy (SEM) was used to determine pre‐ and postexposure architecture of biofilms. The mechanism of the antibiofilm activity of curcuminoids‐LDH was determined using UV‐visible spectroscopy. All tested bacteria had given a zone of inhibition in the presence of curcuminoids‐LDH. The MIC values were 0.200 g/ml for *P*. *aeruginosa*, 0.025 g/ml for *S*. *aureus*, and 0.100 g/ml for *E*. *faecalis*. The 48 hr matured biofilms were reduced by curcuminoids‐LDH with an MBIC_50_ of 0.100 g/ml. The minimum time to achieve MBIC_50_ was 3 hr, and the reduction was constant until 48 hr. SEM images showed a significant reduction of biofilm cell density and exopolymer matrics for all biofilms in the presence of curcuminoids‐LDH. UV‐visible studies revealed the antibiofilm activity of curcuminoids‐LDH as due to the auto‐oxidation of curcuminoids. The oxidation products are more limited in both product concentration per unit time and the variety of products, compared to pure curcuminoids, resulting in sharper UV‐visible peaks than in the case of the latter. Curcuminoids‐LDH has a potential antibacterial activity against *P*. *aeruginosa*,* S*. *aureus*, and *E*. *faecalis*. An antibiofilm activity has been achieved within 3 hr of the treatment. Curcuminoids released from the LDH showed the antibacterial activity due to oxidation products interfering with bacterial cell functions, and also encapsulation in the LDH causes curcuminoids to exhibit the activity in a persistent manner compared to pure curcuminoids.

## 1 INTRODUCTION

Microbial resistance to antibiotics is an emerging fact that is leading to treatment failures (Jones et al., 2008; Simoes, 2011). In chronic infections, antibiotic resistance is due to changes in bacterial growth mode from free‐swimming planktonic cells into sessile communities of structured biofilms. Bacterial biofilms are usually protected within a self‐produced extracellular polymeric substance (EPS) consisting of exopolysaccharide, deoxyribonucleic acid (DNA), and lipids (Ishida et al., 1998). These biofilms show extreme resistance to most conventional antibiotics. Therefore, new approaches have been emerging for the discovery of new drugs with broad‐spectrum of activity.

Curcumin (1,7‐bis(4‐hydroxy‐3‐methoxyphenyl)‐1,6‐heptadiene‐3,5‐dione), a natural poly‐phenolic pigment from *Curcuma longa* (turmeric) rhizomes, is a well‐known antimicrobial agent (Soheil et al., 2014). Poor solubility and bioavailability of curcumin in aqueous solution limit the use of curcumin in medical and clinical therapeutic applications (Hatcher, Planalp, Cho, Torti, & Torti, 2008). Many studies have been reported on modified curcumin with different functionalities, for evaluation for purposes of increasing curcumin solubility and bioavailability, such as encapsulation of curcumin in liposomes, polymeric micelles, intercalation in cyclodextrins (Naksuriya, Okonogi, Schiffelers, & Hennink, 2014; Yallapu, Jaggi, & Chauhan, 2012).

Layered double hydroxide (LDH) nanocomposites are a class of inorganic material with a unique structure of outer positively charged metal hydroxide sheets and inner interlayer anions, hydrated with water molecules aiding in its uptake and cellular penetration (Del, 2007; Indrasekara & Kottegoda, 2011; Karunaratne, Kottegoda, & De Alwis, 2012; Kovanda et al., 2009; Wang & O'Hare, 2012). A controllable anion exchange that is pH dependent is possible due to the fascinating structure of LDH (Del, 2007; Megalathan et al., 2016). Its controlled‐release properties make it a valuable choice for biological and pharmaceutical applications. In the fields of biomedical applications, different types of LDH have been exploited for drug delivery, which have reached an advanced level of clinical trials and use because of their local sustained release and high intrinsic pharmacological activity compared with other conventional drugs (Ladewig, Niebert, Xu, Gray, & Lu, 2010; Ladewig, Xu, & Lu, 2009; Megalathan et al., 2016; Perera, Weerasekera, & Kottegoda, 2015). One such study is an encapsulation of natural curcuminoids to layered double hydroxides (Samindra & Kottegoda, 2014). To the best of authors’ knowledge, only one study has been reported on the antimicrobial activity of encapsulated curcuminoids (Megalathan et al., 2016). However, no study has been conducted to evaluate either in vitro or in vivo antibiofilm activity of curcuminoids‐intercalated LDHs.

This study is focused on studying the in vitro model of antibacterial and antibiofilm activity of selectively encapsulated natural curcuminoids‐LDH toward free‐floating planktonic cells, and mono‐ and cobiofilms of three different selected bacterial species, *Staphylococcus aureus*,* Pseudomonas aeruginosa*, and *Enterococcus faecalis*.

## 2 MATERIALS AND METHODS

### 2.1 Chemicals

The rhizomes of *Curcuma longa* were purchased from an Ayurvedic Pharmacy, Navinna, Sri Lanka. All chemicals used for the synthesis of curcuminoids‐LDH were of analytical grade and procured from Research Lab Fine Chem Industries and Lobachemie Chemicals.

Brain Heart Infusion Broth (BHI) [Oxoid, UK], Nutrient agar [HiMedia, India], and Muller Hinton agar (MHA) [HiMedia, India] were the bacteriological culture media used for the experiments. Phosphate buffered saline (PBS) was used to wash harvested bacterial cells. Working solution concentrations were made by diluting a stock surfactant solution using sterile distilled water.

### 2.2 Synthesis of curcuminoids‐intercalated LDH

#### 2.2.1 Extraction of natural antimicrobial agents

Curcuminoids were extracted from turmeric rhizomes according to the methodology described by Samindra and Kottegoda (2014). The powder of dried turmeric rhizomes (20.0 g) was extracted using a Soxhlet extractor, with hexane (100.0 ml) for 2 hr to remove any oily substances, then re‐extracted with acetone (250.0 ml) for 6 hr. The extracted portion was allowed to solidify by solvent evaporation.

#### 2.2.2 Intercalation of curcuminoids into LDH

The encapsulation was carried out by using the in situ selective encapsulation method described by Megalathan et al. (2016). A solution of 0.100 mol of Mg((NO_3_)_2_.6H_2_O and 0.050 mol of Al(NO_3_)_3_.9H_2_O in 250.00 ml of distilled water was added drop wise to 100 ml of a concentrated (0.4 g/ml) solution of crude turmeric in an acetone/water mixture (1:1) under a nitrogen atmosphere and with vigorous stirring, until a precipitate formed. A solution of 1.0 mol/dm³ NaOH was used to maintain the pH at 10. The precipitate was separated, washed thoroughly with distilled water, and dried at 90°C.

### 2.3 Preparation of samples for antibacterial applications

Curcuminoids‐LDH suspensions of desired concentrations were prepared using distilled water by shaking with distilled water while adding concentrated acetic acid until a pH of 3 was reached. For the working suspension, dried curcuminoids‐LDH (0.2 g) was suspended in distilled water while adding drops of 1 M of acetic acid to acidify the suspensions until a pH of 3 was reached.

### 2.4 Strains and culture conditions

*Pseudomonas aeruginosa* (ATCC 27853), *Staphylococcus aureus* (ATCC 25923), *Enterococcus faecalis* (ATCC 29212), and a total of nine clinical isolates (*P*. *aeruginosa* DCW 12A, DCW 37C and DCW 46A, *S*. *aureus* DCW 11B, DCW 35D and DCW 41B, *E*. *faecalis* DCW 4B, DCW 26E, and DCW 45E) were tested in the study. These bacterial species were subcultured from freezer stocks onto freshly prepared and quality controlled nutrient agar plates and incubated at 37°C for 24 hr. For all planktonic and biofilm assays, BHI [Oxoid, UK] broth was used as the culture medium.

### 2.5 Determination of the effect of curcuminoid*s*‐LDH on planktonic bacteria cells

The effect of curcuminoids‐LDH on planktonic *P*. *aeruginosa*,* S*. *aureus*, and *E*. *faecalis* cells (for both the standard strains and the clinical isolates) was determined by the agar well diffusion method (Magaldi et al., 2004). For each tested bacterial strain, a suspension was prepared by dissolving a portion of their isolated colony in sterile normal saline and turbidity was adjusted to the McFarland 0.5 turbidity standard.

The prepared cell suspensions were inoculated separately using a sterile cotton swab on to the prepared quality controlled MHA plates separately, in order to get a confluent growth. Using a sterile cork‐borer, four 9 mm wells were cut out of each MHA plate. The bottoms of the wells were sealed by adding a drop of molten agar into the wells using a sterile pipette. Aliquots (200 μl) of each of the working solutions of prepared curcuminoids‐LDH were filled into the wells separately. Sterile distilled water and LDH itself were used as negative controls for each test. Gentamicin and vancomycin (30 mg/L) were used as positive controls for gram‐negative bacteria and gram‐positive bacteria, respectively. The plates were kept outside for nearly 10 min and incubated aerobically at 37°C. The zones of inhibition were measured after overnight incubation. The presence of any zone of inhibition was considered as an indication of sensitivity to the relevant treatment. Each test was done in triplicate and the average zone of inhibition was calculated.

### 2.6 Determination of minimum inhibitory concentration (MIC) of Curcuminoids‐LDH toward planktonic bacterial cells

Minimum inhibitory concentrations of curcuminoids‐LDH toward planktonic *P*. *aeruginosa*,* S*. *aureus*, and *E*. *faecalis* (for both the standard strains and the clinical isolates) were determined by following the CLSI M‐27A broth microdilution method with modifications (Balouiri, Sadiki, & Ibnsouda, 2016). Standard cell suspensions of bacterial species tested were prepared in sterile BHI broth using the harvested bacterial cells from the 24 hr old liquid bacterial cultures, followed by washing the cells three times using sterile PBS.

Bacterial cell suspensions equal to the 0.5 McFarland turbidity standard were prepared in sterile BHI broth, using the washed cells. Samples of 100 μl from the prepared bacterial cell suspensions were then inoculated in triplicate to a sterile 96‐well polystyrene microtiter plate, and 100 μl of each dilution of nanoparticles was added to each well. After incubation for 24 hr at 37°C, 20 μl from each well was subcultured on fresh nutrient agar plates in order to determine the complete inhibition of the growth of the tested bacterial cells.

### 2.7 Determination of minimum biofilm inhibitory concentration (MBIC_50_)

In order to prepare standard cell suspensions of *P*. *aeruginosa*,* S*. *aureus*,* E*. *faecalis*, and their 1:1 mixed suspensions, the turbidity of cell suspensions in BHI broth was adjusted to the 0.5 McFarland turbidity standard. A constant volume (100 μl) of prepared suspensions was inoculated into the 96 sterile wells in a flat bottomed microtiter plate. The plate was sealed with para‐film and incubated at 37°C for 48 hr. The culture medium was replenished with the same volumes of BHI broth at 24 hr. The mature biofilms, including monospecies biofilms of *P*. *aeruginosa*,* S*. *aureus*, and *E*. *faecalis* (for both the standard strains and the clinical isolates), and their 1:1 mixed species cobiofilms (only for the standard strains) were washed twice with sterile PBS.

Sterilized BHI broth (100 μl) and hundred microliters of gentamicin (30 mg/L) were added as the positive control, into the corresponding wells. For the treatment of biofilms, sterilized BHI broth (100 μl) was added to each washed well and the corresponding wells were treated with 100 μl of a dilution series of curcuminoids‐LDH ranging from 0.001 to 0.200 g/ml. Further, growth control (cells + broth), media control (only broth), and blank control (broth + curcuminoid‐LDH) were included. The microtiter plate was covered with the lid, sealed with para‐film, and incubated at 37°C for a further 24 hr.

Following overnight incubation, the microtiter plate was washed thrice with 200 μl of sterile PBS and biofilm biomass was determined by using the MTT assay and CV assay. To quantify the viable cell mass, 50 μl of 1 mg/ml MTT aqueous solution was added to each well and incubated at 37°C for 4 hr. After incubation, the remaining MTT solution was aspirated. To each well, 100 μl of dimethyl sulfoxide (Sigma‐Aldrich, USA) was added to dissolve the formazan product and absorbance was measured by using the detection and reference wavelengths at 570 and 630 nm, respectively, using a microtiter plate reader (Thermo fisher Scientific, Multiskan Ex, S/N 3550905398, USA).

For CV assay, 100 μl of 0.1% CV solution was added to each well and incubated for 20 min at 37°C and plate was washed carefully thrice with sterile PBS. Finally, CV stained cells were decolorized by adding 100 μl/well of 30% acetic acid. Then, 50 μl of acetic acid was transferred to a new microtiter plate and absorbance was measured at 595 nm using a microtiter plate reader. MBIC 50 values were calculated with respect to the negative controls of each study identifier using the software Graph Pad Prism 6.0.1.

### 2.8 Determination of minimum killing time for minimum biofilm inhibitory concentration

Matured monospecies biofilms of standard strains and clinical isolates of *P*. *aeruginosa*,* S*. *aureus*, and *E*. *faecalis*, and 1:1 mixed species cobiofilms were grown in vitro in a sterile, 96‐well, flat bottomed microtiter plate. The grown biofilms were washed thrice with 200 μl of sterile PBS, treated with 100 μl of curcuminoids‐LDH, and incubated at 37°C at 3, 6, 12, 18, 24, 36, and 48 hr. Following incubation for the above time periods, the viable cell mass was quantified using the MTT assay along with negative controls as previously described. The time required to reduce the biofilm biomass by 50% of the negative control was obtained as the minimum killing time for minimum biofilm inhibitory concentration.

### 2.9 Scanning electron microscopy (SEM)

Scanning electron microscopy was used to examine the architectural properties of biofilms of *P*. *aeruginosa*,* S*. *aureus*, and *E*. *faecalis* before and after exposure to curcuminoids‐LDH. Briefly, biofilms were grown on sterile coverslips of 10 mm diameter, in BHI medium, for 48 hr (Harriott & Noverr, 2009; Tsang, Bandara, & Fong, 2012). The matured biofilms were treated with curcuminoids‐LDH nanohybrid and incubated for another 24 hr, fixed with 2.5% glutaraldehyde at +4°C for 2 hr, and serially dehydrated with ethanol. After drying in a desiccator, the biofilms were coated with gold and examined using the scanning electron microscope (Hitachi SU 6600) in the secondary electron mode.

### 2.10 UV‐visible spectroscopic analysis

UV‐visible spectroscopy was used to determine the antibiofilm mechanism of curcuminoids‐LDH. For that, the 48 hr matured biofilms were treated with the stock solution of curcuminoids‐LDH (0.200 g/ml). After 24 hr, the supernatant of the biofilm was filtered using a 0.2 μm filter, to remove any solid particles and bacterial cells. UV‐visible spectra were recorded using UV—2602 Labomed, Inc., single beam scanning spectrophotometer in the wave length region of 200–500 nm.

### 2.11 Determination of cytotoxicity

Cytotoxicity of curcuminoids and curcuminoids‐LDH on normal lung cell line MRC‐5 was determined by sulforhodamine B (SRB) assay according to Skehan with minor modifications (Skehan et al., 1990). Cells (5 × 10^3^/well) were plated in 96‐well culture plates and incubated for 24 hr. After that, the culture medium was removed and replaced with fresh medium containing different doses of curcuminoids and curcuminoids‐LDH. The treated plates were then incubated for 24, 48, 72 hr at 37°C with 5% CO_2_. After the incubation period, cells were fixed with trichloro acetic acid (TCA) for 1 hr at 4°C. Cells were then washed and stained with SRB solution (0.4% wt/vol SRB in 1% acetic acid) for 15 min at room temperature. The dye was removed and the cells were rinsed with 1% acetic acid and then the plate was air‐dried. Tris‐base was added to each well, the absorbance values were taken at OD 540 nm, and the results are expressed as percentage cell viability (mean of control group – mean of treated group/control group × 100). IC_50_ values were calculated using the software Graph Pad Prism 6.0.1.

### 2.12 Statistical analysis

The statistical analysis was carried out by using the software, Statistical Package for Social Sciences (SPSS) version 20. Multiple means of more than three data sets were compared using one way ANOVA and two‐way ANOVA. The level of significance was taken at 5% (*p* < 0.05).

## 3 RESULTS AND DISCUSSION

### 3.1 Characterization

The presence of curcuminoids in acetone was confirmed by thin layer chromatography (TLC). The corresponding R_f_ values for curcumin, DMC, and BDMC were 0.75, 0.55, and 0.27, respectively, which was in line with the R_f_ values reported in a previous work (Megalathan et al., 2016).

### 3.2 Effect of curcuminoids‐LDH on planktonic bacteria

The antimicrobial activity of curcuminoids‐LDH was tested against all the standard *P*. *aeruginosa*,* S*. *aureus*, and *E*. *faecalis* strains and their clinical isolates. According to the observations, the extracted curcuminoids showed inhibitory activity against the tested microbial species.

Curcuminoids‐LDH (0.200 g/ml) demonstrated an antimicrobial activity for all tested bacterial strains (Table 1).

**Table 1:** Antibacterial behavior of Curcuminoid‐LDH at pH 3

Tested bacterial species	Zone Inhibition Diameter/mm		
	Curcuminoid‐LDH	NO_3_‐LDH	(+) control
*P*. *aeruginosa* ATCC 27853	28.0 ± 2.5	0	16.5 ± 0.5
*S*. *aureus* ATCC 25923	25.5 ± 1.5	0	16.5 ± 0.5
*E*. *faecalis* ATCC 29212	31.0 ± 2.5	0	16.0 ± 0.0
*P*. *aeruginosa* DCW 12A	27.5 ± 2.6	0	16.5 ± 0.5
*P*. *aeruginosa* DCW 37C	27.3 ± 2.8	0	16.2 ± 0.5
*P*. *aeruginosa* DCW 46A	27.0 ± 2.5	0	16.0 ± 0.8
*S*. *aureus* DCW 11B	25.0 ± 1.5	0	16.5 ± 0.5
*S*. *aureus* DCW 35D	25.6 ± 1.5	0	16.5 ± 0.4
*S*. *aureus* DCW 41B	25.2 ± 1.5	0	16.5 ± 0.5
*E*. *faecalis* DCW 4B	30.4 ± 2.2	0	15.6 ± 0.0
*E*. *faecalis* DCW 26E	30.6 ± 2.4	0	16.0 ± 0.0
*E*. *faecalis* DCW 45E	31.0 ± 2.5	0	15.8 ± 0.0

Note

Vancomycin and gentamicin were used as the positive controls, and the sterile acidic solvent was used as the negative control.

The average zone of inhibition given by curcuminoids‐LDH at pH 3, against all bacterial species, was significantly greater than the average zone of inhibition of the control (*p* < 0.05). This suggests that curcuminoids‐LDH has an improved slow release property against both gram‐positive and gram‐negative bacterial species.

Layered double hydroxides (LDH) nanocomposites are a group of anionic clay‐like materials with unique layered structures. Due to the substitution of trivalent cation Al^3+^ by divalent cation Zn^2+^ or Mg^2+^, the LDH nanoparticles have positively charged sheets. According to the study findings, no zone inhibition was seen in neat LDH intercalated with nitrate ions_._

Under natural pH conditions, the polypeptides of bacteria and viruses are composed of weakly acidic and basic groups (e.g., carboxyl and amino moieties), and the isoelectric point lies in between pH 3 and pH 4. Thus, there is a possibility to remove bacteria by adsorption to pure LDH with lower concentrations of bacterial suspensions such as 1 × 10^4^ (Yang, Chang, & Zhao, 2013). Since LDHs are anionic clay‐like materials, LDH can adsorb negatively charged bacterial cell walls (Van Der Wal, Norde, Zehnder, & Lyklema, 1997). However, it depends on the zeta potential of LDH and varies with the species type that has been intercalated (Yang et al., 2013).

Further, at low concentrations of curcuminoid‐LDH which were around 0.5 × 10^−3^ and fewer concentrations, it was observed that the growth of biofilm was increased rather than an inhibition at pH 3. This indicates that pH 3 could not perform an inhibition itself for the considered bacterial species.

### 3.3 Minimum inhibitory concentration (MIC) of planktonic bacterial cells

According to the CLSI M‐27A broth microdilution method carried out with several modifications, the MIC values of curcuminoids‐LDH were 0.200, 0.025, and 0.100 g/ml for planktonic *P*. *aeruginosa*,* S*. *aureus*, and *E*. *faecalis*, (for both the standard strains and clinical isolates), respectively. The MIC of curcuminoids has been assessed in several studies for several bacterial species, which had smaller MIC values. The MICs have varied from 0.125 to 625 μg/ml for pure curcumin and from 4 to 16 mg/ml for *Curcuma longa* aqueous extract (Neelakantan et al., 2013; Niamsa & Sittiwet, 2009; Packiavathy, Priya, Pandian, & Ravi, 2014). The above change in MICs could occur because curcuminoids release from the curcuminoids encapsulated LDH is a slow and controlled process. Further, the MICs were assessed only for 48 hr in the study. Therefore, it could be one of the reasons for higher the MIC value of curcuminoids‐LDH in the present study.

Based on the experimental data, the order of increase in sensitivity was *S*. *aureus > E*. *faecalis > P*. *aeruginosa*. The results indicated that the selected gram‐positive bacteria had higher sensitivity than the selected gram‐negative bacteria. This could be due to differences in their cell membrane constituents and structure. It is known that gram‐positive bacteria possess a thick outer peptidoglycan layer, while gram‐negative bacteria have a thin outer phospholipidic membrane, both of which undergo different types of interactions with curcumin (Bhawana, Basniwal, Buttar, Jain, & Jain, 2011).

### 3.4 Minimum biofilm inhibitory concentration (MBIC_50_)

All biofilms were inhibited (>50%) by the working concentration of gentamicin (30 mg/L). According to the findings, there was no significant difference between the inhibitory activity of the working concentration of curcumin‐LDH and the control (*p* < 0.05).

The inhibitory concentration of antimicrobial agent required to reduce the biofilm biomass to 50% of the biomass of the negative control (biofilms without antimicrobial treatments) is defined as MBIC_50_ which is measured by antimicrobial assays including MTT and CV methods.

**Figure 1:**
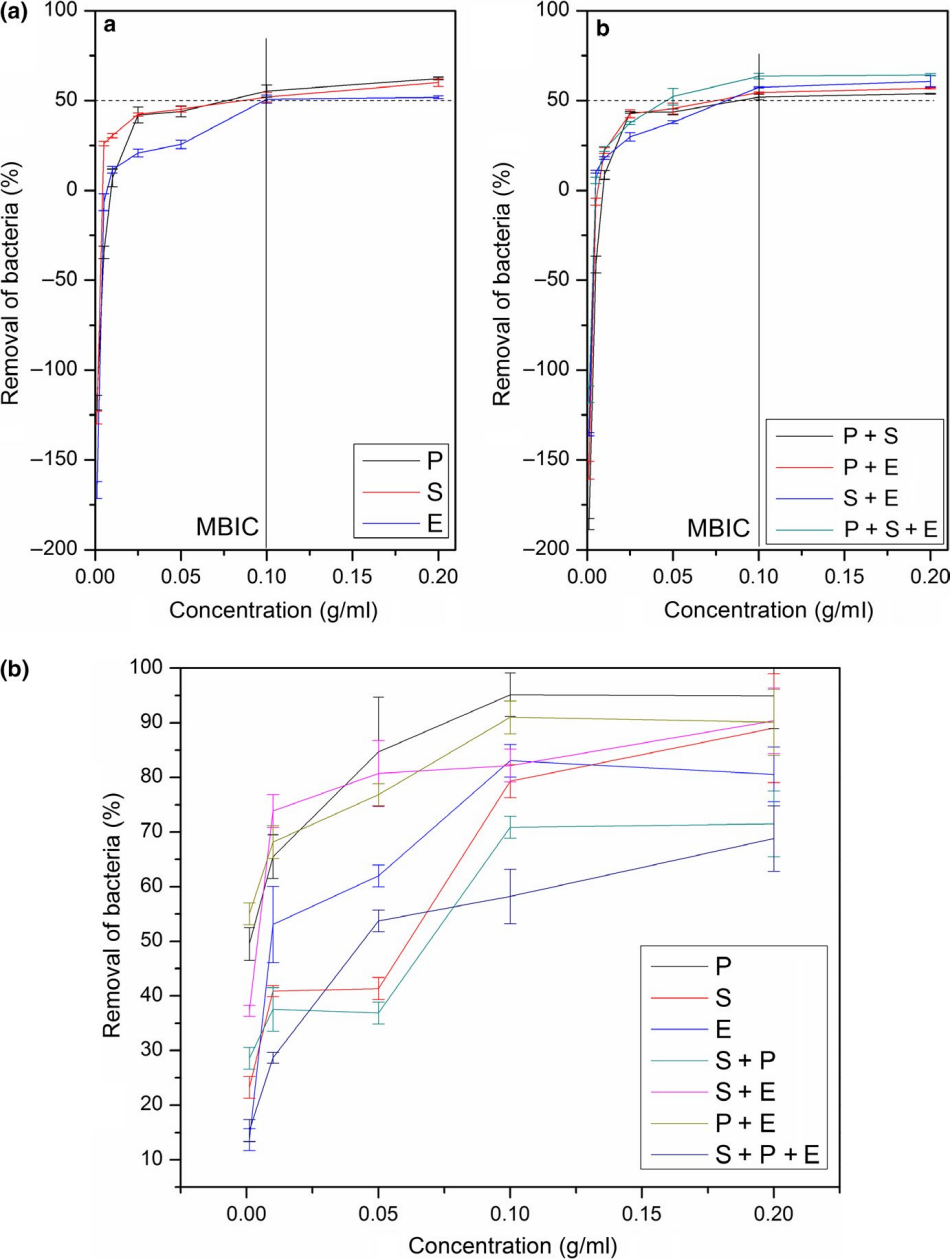
(a) Biofilm Inhibition behavior of curcumin‐LDHs at different concentrations based on MTT assay (For standard bacterial strains of *P*. *aeruginosa* (ATCC 27853), *S*. *aureus* (ATCC 25923), *E*. *faecalis* (ATCC 29212)). P: Monospecies biofilm of *P*. *aeruginosa*. S: Monospecies biofilm of *S*. *aureus*. E: Monospecies biofilm of *E*. *faecalis*. P + S: Dual species biofilm of *P*. *aeruginosa* and *S*. *aureus*. P + E: Dual species biofilm of *P*. *aeruginosa* and *E*. *faecalis*. S + E: Dual species biofilm of *S*. *aureus* and *E*. *faecalis*. P + S + E: Cobiofilm with *P*. *aeruginosa*,* S*. *aureus*, and *E*. *faecalis*. (b) Biofilm Inhibition behavior of curcumin‐LDHs at different concentrations based on CV assay (For standard bacterial strains of *P*. *aeruginosa* (ATCC 27853), *S*. *aureus* (ATCC 25923), *E*. *faecalis* (ATCC 29212)). P: Monospecies biofilm of *P*. *aeruginosa*. S: Monospecies biofilm of *S*. *aureus*. E: Monospecies biofilm of *E*. *faecalis*. P+S: Dual species biofilm of *P*. *aeruginosa* and *S*. *aureus*. P + E: Dual species biofilm of *P*. *aeruginosa* and *E*. *faecalis*. S + E: Dual species biofilm of *S*. *aureus* and *E*. *faecalis*. P + S + E: Cobiofilm with *P*. *aeruginosa*,* S*. *aureus*, and *E*. *faecalis*

The results showed that at least a concentration of 0.010 g/ml of curcuminoids‐LDH was required to destroy the increasing growth of the biofilm, while a concentration of 0.100 g/ml was required for reaching the MBIC_50_ of the monospecies biofilms comprising standard strains of *P*. *aeruginosa*,* S*. *aureus*, and *E*. *faecalis* and their 1:1 mixed species cobiofilms (Figure 1a,b). However, the exact MBIC_50_ was strain dependent, showing their different resistant abilities against curcuminoids‐LDH (Table 2).

**Table 2:** Minimum killing time of curcumin‐LDH for biofilms of standard ATCC bacterial strains

Biofilm	Minimum killing time of MBIC_50_
*P*. *aeruginosa* + *S*. *aureus* + *E*. *faecalis*	5 hr 45 min
*P.aeruginosa* + *E*. *faecalis*	17 hr 30 min
*P*. *aeruginosa*	8 hr 15 min
*S*. *aureus* + *E*. *faecalis*	20 hr 00 min
*S*. *aureus*	26 hr 30 min
*P*. *aeruginosa* + *S*. *aureus*	14 hr 30 min
*E*. *faecalis*	20 hr 30 min

Note

*P*. *aeruginosa* (ATCC 27853), *S*. *aureus* (ATCC 25923), *E*. *faecalis* (ATCC 29212).

**Figure 2:**
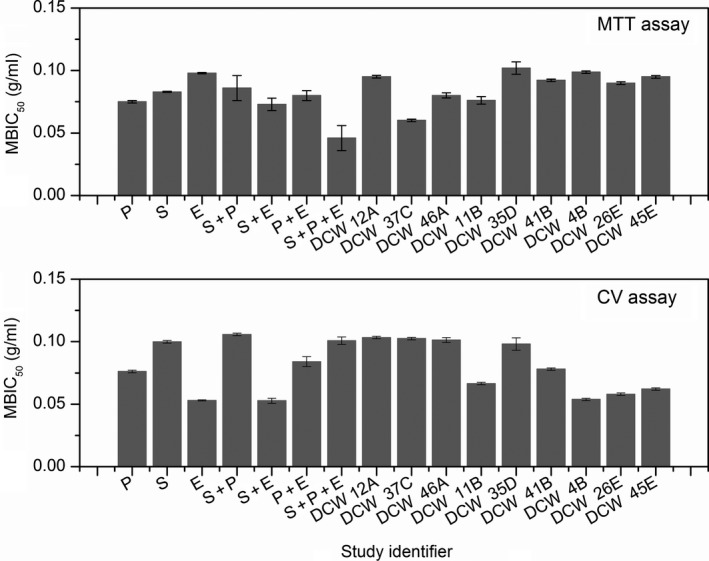
Biofilm Inhibition behavior of Curcumin‐LDHs (MBIC_50_) on both the standard strains and the clinical isolates, at different concentrations based on MTT and CV assays. (P: *P*. *aeruginosa* (ATCC 27853), S: *S*. *aureus* (ATCC 25923), E: *E*. *faecalis* (ATCC 29212)). P: Monospecies biofilm of *P*. *aeruginosa*. S: Monospecies biofilm of *S*. *aureus*. E: Monospecies biofilm of *E*. *faecalis*. P + S: Dual species biofilm of *P*. *aeruginosa* and *S*. *aureus*. P + E: Dual species biofilm of *P*. *aeruginosa* and *E*. *faecalis*. S + E: Dual species biofilm of *S*. *aureus* and *E*. *faecalis*. P + S + E: Cobiofilm with *P*. *aeruginosa*,* S*. *aureus*, and *E*. *faecalis*. Other numbers are their clinical isolates

When considering the monospecies biofilms of all nine clinical isolates, they have shown that their MBIC_50_ has been achieved prior to the concentration of 0.100 g/ml is reached. This finding was in line with the findings of their standard monospecies biofilms (Figure 2).

The formation of biofilm plays an important role in pathogenesis, and the development of biofilm is based on the signal‐mediated quorum sensing system (Jones et al., 2008). In the recent study done by Packiavathy et al. (2014), a reduced production of biofilm biomass was found in uropathogens when treated with curcumin, suggesting that curcumin may interfere with quorum sensing system, and that may prevent the development of bacterial biofilms (Packiavathy et al., 2014). To provide a better understanding of the effect of biofilms is necessary for the correlation between the results of CV and the MTT assays. Although the CV assay serves as an indicator of adhesion, it does not reveal the metabolic status of the cells. MTT assay is a tetrazolium salt, which in the presence of metabolically active cells is reduced into a product that can be measured colorimetrically, serving as a respiratory indicator of live cells (Krom, Cohen, Feser, & Cihlar, 2007). In biofilms, the MTT assay was used as an indicator of attached viable cells, while CV stains both viable and nonviable cells that may be attached (Kouidhi, Zmantar, Hentati, & Bakhrouf, 2010). Both methods showed a good antibiofilm activity of curcumin‐LDH. Further, they have mentioned that curcumin can reduce not only the biomass but also microcolony formation. Further, consistent with the findings of Packiavathy et al., curcumin has been shown to inhibit the biofilm of *P*. *aeruginosa* without affecting the planktonic growth (Rudrappa & Bais, 2008).

### 3.5 Minimum killing time of MBIC_50_ for 48 hr mature biofilms

In the minimum killing time of the minimum biofilm inhibitory concentration assay, the 48 hr mature monospecies and 1:1 mixed species cobiofilms of only three standard bacterial strains were exposed to curcuminoids‐LDH, antimicrobial nanohybrid solutions in sterile BHI culture medium, at MBIC_50_ up to 48 hr.

**Figure 3:**
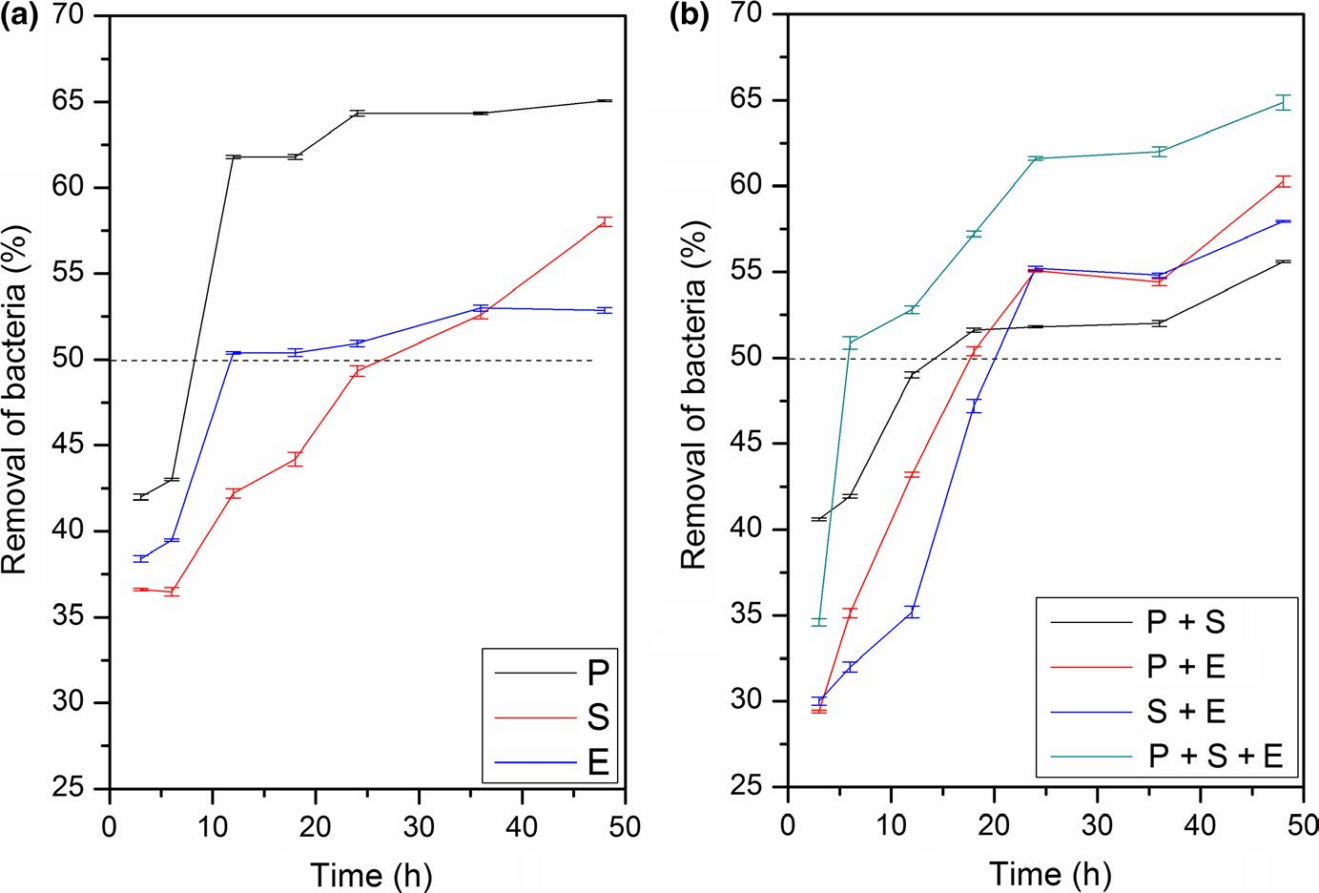
Minimum killing time of the minimum biofilm inhibitory concentration of curcumin‐LDH over 48 hr for different monospecies and mixed species biofilms of standard bacterial strains [*P*. *aeruginosa* (ATCC 27853), *S*. *aureus* (ATCC 25923), *E*. *faecalis* (ATCC 29212)]. P: Monospecies biofilm of *P*. *aeruginosa*. S: Monospecies biofilm of *S*. *aureus*. E: Monospecies biofilm of *E*. *faecalis*. P + S: Dual species biofilm of *P*. *aeruginosa* and *S*. *aureus*. P + E: Dual species biofilm of *P*. *aeruginosa* and *E*. *faecalis*. S + E: Dual species biofilm of *S*. *aureus* and *E*. *faecalis*. P + S + E: Cobiofilm with *P*. *aeruginosa*,* S*. *aureus*, and *E*. *faecalis*

The time taken to achieve MBIC_50_ was significantly different with the biofilm type, confirming the varying resistance abilities of the different biofilms. The minimum killing time to achieve MBIC_50_ of 48 hr matured mono‐ and cocultured biofilms was in an average time period of 6 to 27 hr (Figure 3).

At pH 3, with the medium highly protonated, the release of curcuminoid ions from the layered structure to the medium fits the zero kinetic model (Megalathan et al., 2016). The zero kinetic model describes the slow release behavior of low soluble drugs and their prolonged releasing ability, which ideally suits the low water soluble curcuminoids in the LDH sandwich (Costa & Lobo, 2001). Also, equilibrium conditions which may cause considerable changes in the reduction of biofilms were not applicable to such kinetic systems, which result in prolonged constant killing ability. Therefore, it can be concluded that curcuminoids have been released slowly, making the minimum killing time longer. Also, the LDH shield has protected curcuminoids from degradation over 48 hr, although 60% of pure curcumin can degrade within 25 min (Manju & Sreenivasan, 2011). However, further studies are required in order to estimate the maximum time during which the prolonged antibiofilm activity of curcumin‐LDH can be maintained.

### 3.6 Scanning electron microscopy (SEM)

Based on the findings of biofilm susceptibility testing, SEM was performed on 48 hr mature *P*. *aeruginosa*,* S*. *aureus*, and *E*. *faecalis* monospecies biofilms, and 1:1:1 mixed species cobiofilm treated with curcumin‐LDH in order to determine the postexposure architectural changes of biofilms and also to strengthen the study findings.

**Figure 4:**
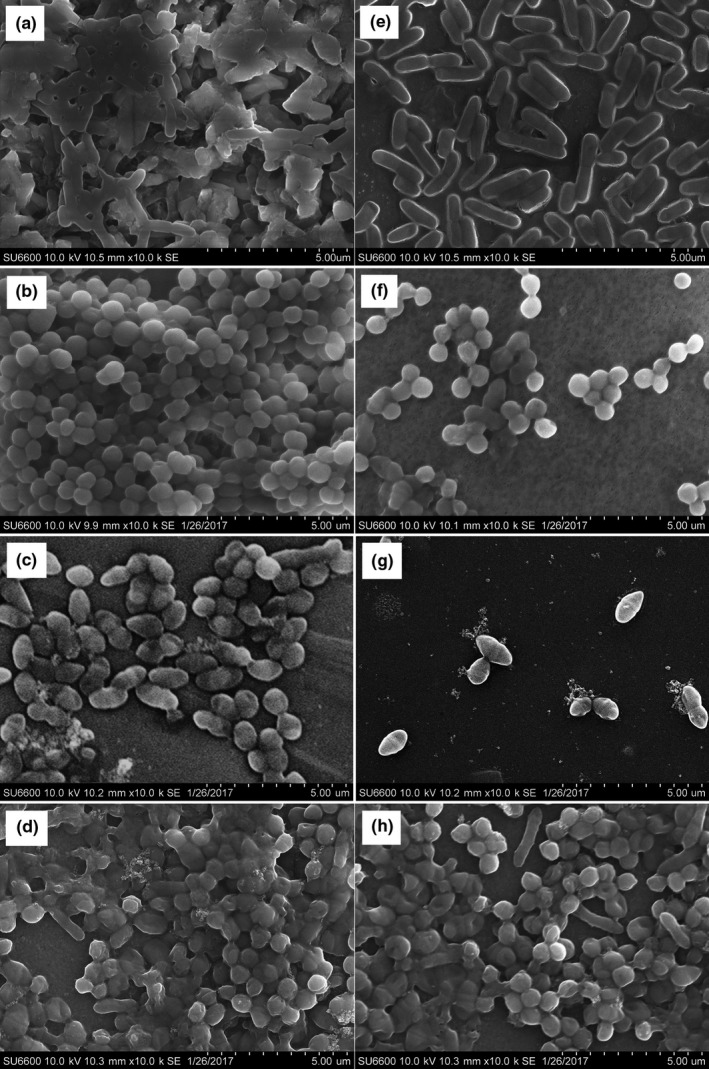
SEM images of mono and mixed species biofilms of standard bacterial strains [*P*. *aeruginosa* (ATCC 27853), *S*. *aureus* (ATCC 25923), *E*. *faecalis* (ATCC 29212)]. (a–c) Before the treatment for monospecies biofilm of *P*. *aeruginosa*,* S*. *aureus*, and *E*. *faecalis*, respectively. (d) Before the treatment for cobiofilm with *P*. *aeruginosa*,* S*. *aureus*, and *E*. *faecalis*. (e–g) After the treatment for monospecies biofilm of *P*. *aeruginosa*,* S*. *aureus*, and *E*. *faecalis*, respectively. (f) Before the treatment for cobiofilm with *P*. *aeruginosa*,* S*. *aureus*, and *E*. *faecalis*.)

The antibiofilm activity of curcumin is caused by the inhibition of the development of biofilm at the initiation and at the maturation stages. Curcumin interferes with quorum sensing, which controls the development of biofilms and thereby reduces microcolony formation (Balouiri et al., 2016). In this study, a significant reduction of biofilm cell density and the amount of extracellular polymeric substances (EPS) were observed for all 48 hr matured biofilms in the presence of curcuminoids‐LDH (Figure 4). The reduction of EPS tends to reduce an additional source of nutrients for further cell growth and a conditioning surface for further attachment. Moreover, the reduction of EPS may have a role in preventing further biofilm formation, since EPS is a quorum sensing dependent factor (Soheil et al., 2014). This finding provides evidence for the growth inhibitory effect of curcumin‐LDH throughout the experiments.

### 3.7 UV‐visible spectroscopic analysis

UV‐visible spectroscopic data were used to obtain evidence regarding the mechanism of the antibacterial activity of curcumin‐LDH in BHI medium.

**Figure 5:**
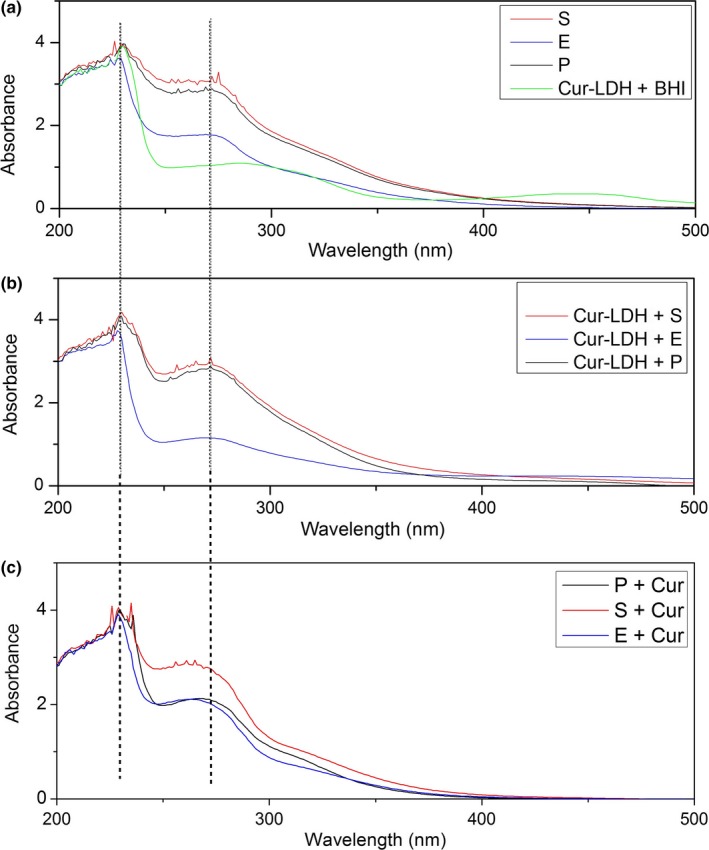
UV‐visible spectra for supernatant of the biofilm in BHI medium; (a) before the antibiofilm treatment; (b) after the treatment with curcumin‐LDH; (c) after the treatment with curcuminoids (P: Monospecies biofilm of *P*. *aeruginosa* (ATCC 27853), S: Monospecies biofilm of *S*. *aureus* (ATCC 25923). E: Monospecies biofilm of *E*. *faecalis* (ATCC 29212))

In aqueous solutions, curcuminoids exhibit absorbance bands in the UV/visible region, with λ_max_ at around 266 nm and at around 426 nm (El Khoury, Abiad, Kassaify, & Patra, 2015). However, the maximum absorbance wavelength depends on the nature of the curcuminoid molecule and has a strong solvent matrix dependency (Zsila, Bikádi, & Simonyi, 2003). Thus, the UV‐visible spectrum for curcuminoids in BHI medium (Figure 5a) has shifted to higher wavelength range, with λ_max_ appearing at around 286 nm and at around 443 nm. In addition, the BHI medium itself provides spectral absorbance in the range 250–275 nm due to various nutritious matrix components such as glucose (two peaks with λ_max_ around 210 and 270 nm) and a series of proteins (λ_max_ around 259) (Kaijanen, Paakkunainen, Pietarinen, Jernström, & Reinikainen, 2015; Trivedi, Patil, Mishra, & Jana, 2015).

After the 24 hr curcuminoids‐LDH treatments of matured biofilms, the absorbance peaks at around 270 nm have right shifted, while the intensities have reduced in the UV‐visible spectra in Figure 5b. This indicates that structural changes have occurred in the solution phase after the inhibition of biofilms, by curcuminoids being released from the LDH shield. The λ_max_ ranges of curcuminoids deviate upon numerous reactions and the subsequent changes in the chemical structure. According to the study done by Gordon et al., the λ_max_ value of curcuminoid oxidation products shows that the actual oxidation product of curcuminoids is a spiroepoxide, with an absorbance maximum at 263 nm. The transformation of curcuminoids into this spiroperoxide happens through a number of intermediates, including radicals and electrophilic intermediates, which interact with bio molecules essential for bacterial proliferation, including DNA and proteins, thus resulting in the inhibition of the culture (Gordon, Luis, Sintim, & Schneider, 2015). Therefore, it is demonstrated that the antibiofilm activity of these systems is basically caused by the auto‐oxidation of curcuminoids, although the exact mechanism of the antibacterial activity of curcumin‐LDH cannot be determined due to interference from the BHI medium matrix.

The other significant observation of the UV‐visible spectrophotometric study is the prominent dissimilarity between the spectra of curcuminoids‐LDH treated biofilm culture media and culture media treated with pure curcuminoids (Figure 5c). The broadening of the peaks in the range 250–300 nm in the latter elaborates the fact that, in contrast with curcuminoids released from the LDH, pure curcuminoids give rise to many oxidation products, resulting in the absorption of energy within a broad range of wavelengths. Thus, the so‐called difference between the spectra could be explained by the sustained release behavior of curcuminoids‐LDH compared to pure curcuminoids. Pure curcuminoids, due to the immediate exposure to external factors like radiations and oxygen in bulk amounts, may undergo oxidation reactions, giving rise not only a majority of oxidation and degradation products but also a number of different chemical species which absorb in a broad range of wavelengths (Gordon et al., 2015). However, the release of curcuminoids in a sustained manner means that only a small number of molecules are exposed to external conditions at any given time. Thus, curcuminoids released from LDH yield oxidation products limited in both parameters, the product concentration per unit time as well as the variety among the products (Gordon et al., 2015). Thus, the LDH intercalation allows the antibiofilm activity of curcuminoids to persist during an extended period of time, compared to pure curcuminoids.

### 3.8 Cytotoxicity of curcuminoids and curcuminoids‐LDH

**Figure 6:**
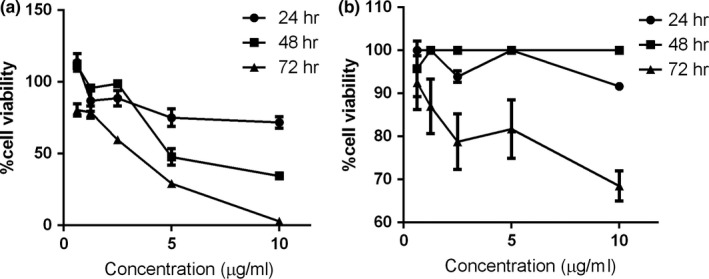
Cytotoxic effect on MRC‐5 cells. (a) Cell viability after exposing to curcuminoids. (b) Cell viability after exposing to curcuminoids‐LDH

Cytotoxicity of curcuminoids and curcuminoids‐LDH toward mammalian cells was determined by exposing the normal lung cell line MRC‐5 to the compounds. Curcuminoids show relatively high cytotoxicity compared to curcuminoids‐LDH where the cytotoxic effect of curcuminoids‐LDH onsets later at 72 hr and is more than 17 times lower than the LD50 of curcuminoids (Table 3, Figure 6a,b).

**Table 3:** IC 50 values of curcuminoids (Cur) and curcuminoids‐LDH (Cur‐LDH) on MRC‐5 cells. Values were calculated at 24, 48, and 72 hr intervals from the results obtained from the SRB assay

Compound	IC 50 (μg/ml)		
	24 hr	48 hr	72 hr
Cur	22.5	6.1	2.8
Cur‐LDH	–	–	48.9

## 4 CONCLUSIONS

The curcuminoids‐LDH has a potential antibacterial activity against *P*. *aeruginosa*,* S*. *aureus*, and *E*. *faecalis*. An antibiofilm activity against their matured single and mixed species biofilms has been achieved within 3 hr of the treatment. The curcuminoids released from the LDH show the antibacterial activity due to the oxidation products interfering with bacteria cell functions, and encapsulation in LDH causes curcuminoids to exhibit this activity in a persistent manner, compared to the pure curcuminoids. Together with significantly lowered cytotoxicity, current results establish the potential of this system to be extended to other pharmaceuticals.

## CONFLICT OF INTEREST

The authors declare that they have no conflict of interest.

## AUTHORS’ CONTRIBUTION

BG and AD carried out bench work, data collection, results analysis, and drafted the manuscript. GKW and SK carried out bench work and involved in manuscript preparation. GA supported for the SEM imaging. SRS and ICP designed and carried out the cytotoxicity studies. MW designed and guided the experiments in antimicrobial studies and finalized the manuscript. NK designed, guided the project, and supported for SEM imaging. All authors read and approved the final manuscript.

## Data Availability

All the data gathered from the study were included in the tables and figures in the manuscript. No additional data are available.
